# Multicenter randomized clinical trial of endovascular treatment for acute ischemic stroke. The effect of periprocedural medication: acetylsalicylic acid, unfractionated heparin, both, or neither (MR CLEAN-MED). Rationale and study design

**DOI:** 10.1186/s13063-020-04514-9

**Published:** 2020-07-14

**Authors:** Vicky Chalos, Rob A. van de Graaf, Bob Roozenbeek, Adriaan C. G. M. van Es, Heleen M. den Hertog, Julie Staals, Lukas van Dijk, Sjoerd F.M. Jenniskens, Robert J. van Oostenbrugge, Wim H. van Zwam, Yvo B.W.E.M. Roos, Charles B.L.M. Majoie, Hester F. Lingsma, Aad van der Lugt, Diederik W.J. Dippel, on behalf of the MR CLEAN-MED investigators, Diederik Dippel, Aad van der Lugt, Bob Roozenbeek, Vicky Chalos, Rob van de Graaf, Wouter van der Steen, Adriaan van Es, Jonathan Coutinho, Bart Emmer, Inger de Ridder, Wim van Zwam, Bart van der Worp, Rob Lo, Koos Keizer, Rob Gons, Lonneke Yo, Jelis Boiten, Ido van den Wijngaard, Jeanette Hofmeijer, Jasper Martens, Wouter Schonewille, Jan Albert Vos, Anil M. Tuladhar, Sjoerd Jenniskens, Karlijn de Laat, Lukas van Dijk, Heleen den Hertog, Boudewijn van Hasselt, Paul Brouwers, Emiel Sturm, Michel Remmers, Thijs de Jong, Anouk Rozeman, Otto Elgersma, Maarten Uyttenboogaart, Reinoud P. H. Bokkers, Julia van Tuijl, Issam Boukrab, Julie Staals, Yvo Roos, Charles Majoie, Robert van Oostenbrugge, Peter Rothwell, Andrew Molyneux, Joanna Moschandreas, Daan Nieboer, Gregory del Zoppo, Rick van Nuland, Alida Annechien Postma, René van den Berg, Ludo Beenen, Pieter-Jan van Doormaal, Geert Lycklama, Albert Yoo, Sebastiaan Hammer, Stefan Roosendaal, Anton Meijer, Menno Krietemeijer, Anouk van der Hoorn, Dick Gerrits, Ben Jansen, Sanne Manschot, Henk Kerkhoff, Peter Koudstaal, Hester Lingsma, Olvert Berkhemer, Adriaan Versteeg, Lennard Wolff, Jiahang Su, Hugo ten Cate, Moniek de Maat, Samantha Donkel, Heleen van Beusekom, Aladdin Taha, Kilian Treurniet, Sophie van den Berg, Natalie LeCouffe, Robert-Jan Goldhoorn, Wouter Hinsenveld, Anne Pirson, Lotte Sondag, Manon Kappelhof, Rik Reinink, Manon Tolhuisen, Josje Brouwer, Sabine Collette, Simone Uniken Venema, Susan Olthuis, Floor Pinkaers, Martin Sterrenberg, Naziha El Ghannouti, Sabrina Verheesen, Rita Sprengers, Wilma Pellikaan, Yvonne Drabbe, Joke de Meris, Michelle Simons, Hester Bongenaar, Anja van Loon, Eva Ponjee, Rieke Eilander, Suze Kooij, Marieke de Jong, Esther Santegoets, Friedus van der Minne, Leontien Heiligers, Yvonne Martens

**Affiliations:** 1 Department of Neurology, Erasmus MC University Medical Center, Rotterdam, The Netherlands; 2 Department of Radiology & Nuclear Medicine, Erasmus MC University Medical Center Rotterdam, Rotterdam, The Netherlands; 3 Department of Public Health, Erasmus MC University Medical Center, Rotterdam, The Netherlands; 4 Department of Neurology, Isala, Zwolle, The Netherlands; 5 Department of Neurology, Cardiovascular Research Institute Maastricht, Maastricht University Medical Center, Maastricht, The Netherlands; 6 Department of Radiology & Nuclear Medicine, HagaZiekenhuis, Radiology, Den Haag, The Netherlands; 7 Department of Radiology & Nuclear Medicine, Radboud University Medical Center, Nijmegen, The Netherlands; 8 Department of Radiology & Nuclear Medicine, Cardiovascular Research Institute Maastricht, Maastricht University Medical Center, Maastricht, The Netherlands; 9 Department of Neurology, Amsterdam UMC, University of Amsterdam, location AMC, Amsterdam, The Netherlands; 10 Department of Radiology & Nuclear Medicine, Amsterdam UMC, University of Amsterdam, location AMC, Amsterdam, The Netherlands

**Keywords:** Ischemic stroke, Acetylsalicylic acid, Heparin, Cerebrovascular disorders, Randomized controlled trial, Endovascular treatment, Thrombectomy, Periprocedural

## Abstract

**Background:**

Despite evidence of a quite large beneficial effect of endovascular treatment (EVT) for ischemic stroke caused by anterior circulation large vessel occlusion, many patients do not recover even after complete recanalization. To some extent, this may be attributable to incomplete microvascular reperfusion, which can possibly be improved by antiplatelet agents and heparin. It is unknown whether periprocedural antithrombotic medication in patients treated with EVT improves functional outcome. The aim of this study is to assess the effect of acetylsalicylic acid (ASA) and unfractionated heparin (UFH), alone, or in combination, given to patients with an ischemic stroke caused by an intracranial large vessel occlusion in the anterior circulation during EVT.

**Methods:**

MR CLEAN-MED is a multicenter phase III trial with a prospective, 2 × 3 factorial randomized, open label, blinded end-point (PROBE) design, which aims to enroll 1500 patients. The trial is designed to evaluate the effect of intravenous ASA (300 mg), UFH (low or moderate dose), both or neither as adjunctive therapy to EVT. We enroll adult patients with a clinical diagnosis of stroke (NIHSS ≥ 2) and with a confirmed intracranial large vessel occlusion in the anterior circulation on CTA or MRA, when EVT within 6 h from symptom onset is indicated and possible. The primary outcome is the score on the modified Rankin Scale (mRS) at 90 days. Treatment effect on the mRS will be estimated with ordinal logistic regression analysis, with adjustment for main prognostic variables. Secondary outcomes include stroke severity measured with the NIHSS at 24 h and at 5–7 days, follow-up infarct volume, symptomatic intracranial hemorrhage (sICH), and mortality.

**Discussion:**

Clinical equipoise exists whether antithrombotic medication should be administered during EVT for a large vessel occlusion, as ASA and/or UFH may improve functional outcome, but might also lead to an increased risk of sICH. When one or both of the study treatments show the anticipated effect on outcome, we will be able to improve outcome of patients treated with EVT by 5%. This amounts to more than 50 patients annually in the Netherlands, more than 1800 in Europe, and more than 1300 in the USA.

**Trial registration:**

ISRCT, ISRCTN76741621. Dec 6, 2017.

## Background

Despite the quite large beneficial effect of endovascular treatment (EVT) on functional outcome after ischemic stroke, about 50% of treated patients die or remain dependent at 3 months [1]. These unfavorable outcomes may not be attributable to unsuccessful recanalization alone, as approximately one third of patients do not recover even when complete recanalization is reached early after stroke onset [2]. The high risk of a poor outcome after complete recanalization may be partially explained by incomplete microvascular reperfusion (IMR), which is known to negatively affect tissue recovery [3–6]. Two main causes of IMR are (I) the formation of microthrombi embolized from the original proximal thrombus, formed in situ by local platelet activation or induced by the EVT itself through vascular endothelial damage, and (II) the formation of neutrophil extracellular traps (NETs), in which platelets, erythrocytes, and other particles conglomerate [7–10]. Microvascular reperfusion might be restored by counteracting these two processes. First, formation of microthrombi could be reduced by the administration of a platelet aggregation inhibitor, such as acetylsalicylic acid (ASA). Second, contrary to tissue plasminogen activators (tPA), unfractionated heparin (UFH) removes histones from the chromatin fibers that form the core of the NETs, making thrombi with a large proportion of NETs more easily dissolvable [11, 12]. In addition, the anticoagulant effect of UFH is also based on inactivation of factor IIa (thrombin) and factor Xa, after binding to and activating the enzyme inhibitor antithrombin. By inactivating thrombin, heparin prevents fibrin formation and also inhibits thrombin-induced activation of platelets and of factors V and VIII [13]. It is therefore likely that in patients treated with EVT for an intracranial large vessel occlusion, periprocedural administration of ASA and/or UFH could improve microvascular reperfusion. This potentially leads to improved functional outcome. However, periprocedural use of ASA and/or UFH may also increase the risk of symptomatic intracranial hemorrhage (sICH).

There are no randomized controlled trials (RCTs) on the effects of periprocedural treatment with platelet aggregation inhibitors in ischemic stroke patients treated with EVT [14]. The ARTIS trial focused on the effect of acute ASA administration in alteplase eligible patients (patients eligible for EVT were underrepresented). This trial demonstrated that ASA increased the risk of sICH without affecting functional outcome [15]. However, the absolute risk of hemorrhage in this trial of 4.3% was much lower than the 6–7% in the pivotal NINDS rtPA trial [16] and in the SITS MOST registry [17]. The potential benefits in EVT—reduce vessel wall inflammation and microthrombi formation—are much larger than in the ARTIS trial. A number of observational and post hoc studies have investigated the periprocedural use of platelet aggregation inhibitors; in several of them they were used for indications other than acute treatment itself (e.g., prior use of ASA based on comorbidity) [18–24]. The occurrence of sICH in these studies varied between 6 and 17%. ASA use during EVT is not reported in the current stroke management guidelines [25]. Based on the results of the post hoc analysis of the MR CLEAN trial and results from the large observational MR CLEAN registry, periprocedural use of ASA may be a useful and safe adjunct to EVT [21, 24].

Although RCT data on the effect of periprocedural UFH use in ischemic stroke patients treated with EVT are lacking as well [14], several studies have investigated the use of intravenous (IV) UFH during EVT [26–29]. The occurrence of sICH in these studies varied between 5 and 12%. However, the risk of sICH appears to be outweighed by a higher overall chance of good functional outcome, suggesting benefit of administering UFH during EVT. Heparin use during EVT is not reported in the current stroke management guidelines [25]. Nevertheless, UFH is actively being used by some neuro-interventionists during EVT, occasionally also as part of standard care. In the Netherlands, substantial center variability exists regarding the use of UFH as well [30]. This underlines the equipoise about periprocedural heparin use. Moreover, patients had better functional outcomes when treated in Dutch centers that treat more patients with UFH, without an increased risk of sICH.

Based on the pathophysiology of IMR, the theoretical and reported expected benefits, and the reported safety profile of the two antithrombotic drugs to be evaluated, we designed an RCT to evaluate the benefits and risks of ASA and UFH, alone, or in combination, and most importantly, their effect on functional outcome after EVT [14].

### Research question

The primary objective of the multicenter randomized clinical trial of endovascular treatment for acute ischemic stroke—the effect of periprocedural medication (MR CLEAN-MED)—is to assess the effect of periprocedural ASA and UFH, alone, or in combination, on functional outcome at 90 days in patients who undergo EVT for acute ischemic stroke caused by a confirmed intracranial large vessel occlusion in the anterior circulation.

## Methods

### Design

**Fig. 1:**
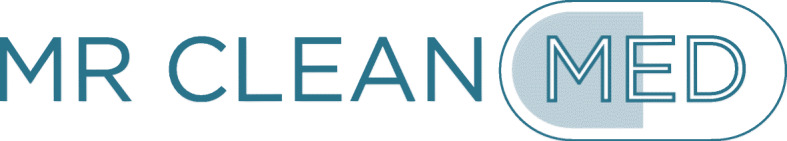
Trial logo

**Fig. 2:**
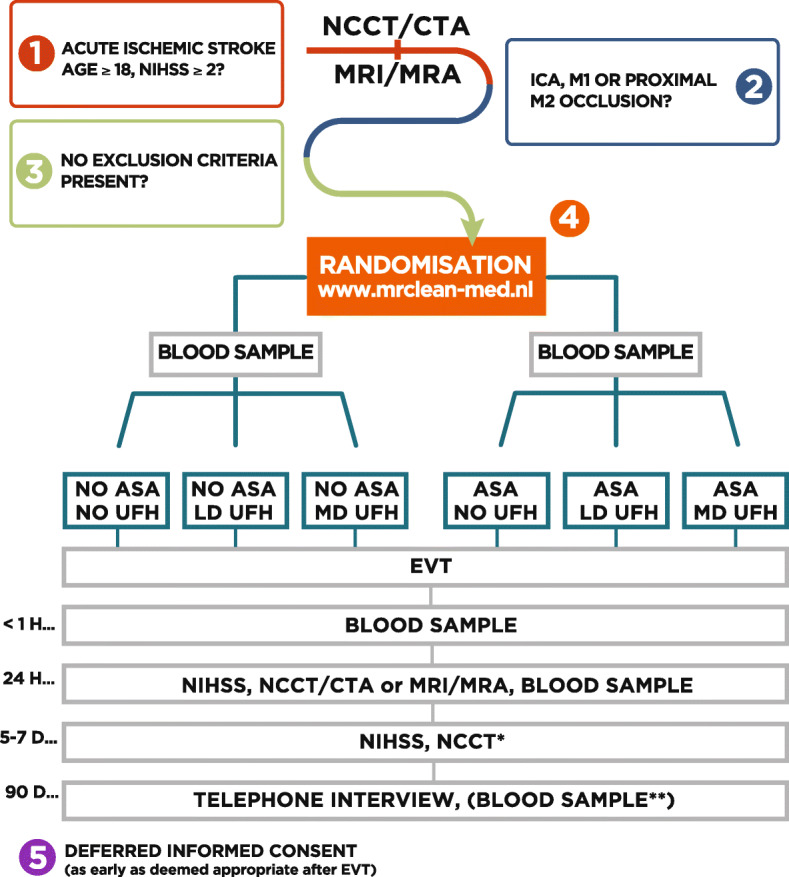
Flow of patients in the MR CLEAN-MED—in first approved protocol version. *Abbreviations:* ASA, intravenous acetylsalicylic acid; CTA, computed tomography angiogram; UFH, intravenous unfractionated heparin; EVT, endovascular treatment; LD, low dose; MD, moderate dose; MRI, magnetic resonance imaging; MRA, magnetic resonance angiography; NCCT, non-contrast computed tomography; NIHSS, National Institutes of Health Stroke Scale. *Captions:* *Only to be performed if imaging at 24 hour was acquired with CT; **Blood sample drawn only in case of regular outpatient clinic appointment within 2-6 months after intervention

**Fig. 3:**
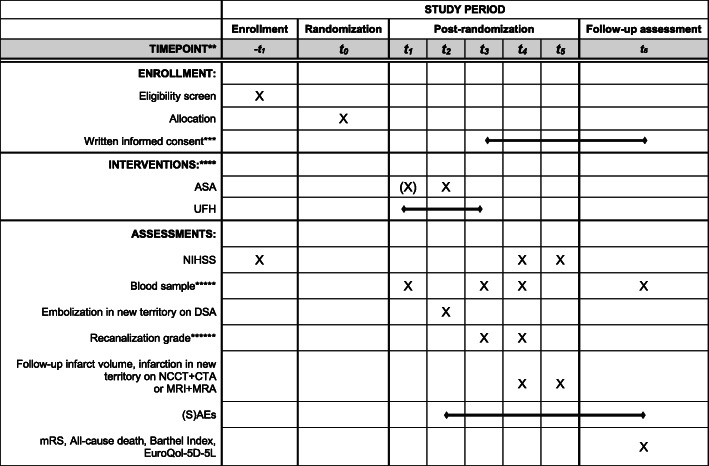
SPIRIT figure *Abbreviations:* MR CLEAN-MED, Multicenter randomized clinical trial of endovascular treatment for acute ischemic stroke. The effect of periprocedural medication: acetylsalicylic acid, unfractionated heparin, both or neither; ASA, intravenous acetylsalicylic acid; UFH, intravenous unfractionated heparin; DSA, digital subtraction angiography; NCCT, non-contract computed tomography; CTA, computed tomography angiography; MRI, magnetic resonance imaging; MRA, magnetic resonance angiography; NIHSS, National Institutes of Health Stroke Scale; (s)ICH, symptomatic intracranial hemorrhage; mRS, modified Rankin Scale.*Captions:* *See trial protocol on https://www.mrclean-med.nl for more information about the patient flow, enrollment, interventions, and assessments; **t_1_ = within 1 hour before groin puncture, after randomization; t_2_ = after groin puncture/during EVT; t_3_ = after EVT; t_4_ = 24 hours after EVT; t_5_ = 5-7 days after EVT or at discharge; t_6_ = 90 days after EVT; ***Informed consent: as early as deemed possible after EVT; ****ASA/UFA loading dose administered directly after groin puncture, or prior to groin puncture in case no recombinant tissue plasminogen activator has been given, UFH continuous infusion until 6 hours after EVT; *****Blood samples: within 1 hour before groin puncture, within 1 hour after EVT, at 24 hours after EVT, if applicable 2-6 months after EVT; ******Recanalization grade on DSA at t_2_, and CTA or MRA at t_4_

MR CLEAN-MED is a multicenter phase III trial with a prospective, randomized, open label blinded end-point (PROBE) design (Fig. 1). Patients are randomized to receive either IV ASA (loading dose only) or IV UFH (low dose or moderate dose, both consisting of a loading dose and continuous infusion for 6 h), both, or neither, as adjunctive treatment to EVT, in a 2 × 3 factorial design. An overview of the treatment arms and main study procedures is provided in Figs. 2 and 3. Patient inclusion started in January 2018.

### Patient inclusion and exclusion criteria

The study population will be drawn from patients with ischemic stroke who enter the emergency department of the EVT center. Patients are eligible for inclusion in the MR CLEAN-MED when they are 18 years or older, have a score of at least 2 on the National Institutes of Health Stroke Scale (NIHSS), present with a clinical diagnosis of acute ischemic stroke, have a non-contrast computed tomography (NCCT) or magnetic resonance imaging (MRI) ruling out intracranial hemorrhage, and have a large vessel occlusion in the intracranial anterior circulation (distal intracranial carotid artery or middle [M1/proximal M2] cerebral artery) confirmed by CT angiography (CTA) or magnetic resonance angiography (MRA). Groin puncture should be possible within 6 h from symptom onset or last seen well. Pretreatment with IV recombinant tissue plasminogen activator (rtPA) according to national guidelines is allowed. Patients already on antiplatelet agents before the index stroke are allowed in the trial.

Exclusion criteria for enrollment in the MR CLEAN-MED are:

- Pre-stroke disability, which interferes with the assessment of functional outcome at 90 days (i.e., pre-stroke modified Rankin Scale [mRS] score > 2);
- Treatment with rtPA, given despite one or more of the following contra-indications: cerebral infarction in the previous 6 weeks with residual neurological deficit or signs of recent infarction on neuroimaging, previous intracerebral hemorrhage within the previous 3 months, INR > 1.7, use of a direct oral anticoagulant (DOAC), rtPA infusion > 4.5 h after symptom onset;
- Contra-indications for ASA or UFH;
- Heparin use in therapeutic dosages that cannot be discontinued;
- INR > 3.0;
- Known hemorrhagic diathesis or known thrombocytopenia (< 90 × 10^9^/L);
- Participation in medical or surgical intervention trials other than the current or Multicentre Randomised trial of Acute Stroke treatment in the Ambulance with a nitroglycerin Patch (MR ASAP, ISRCTN99503308) [31] or A reduction in Time with Electronic Monitoring in Stroke (ARTEMIS, NCT02808806).

### Eligibility criteria for participating centers

Centers should be certified or meet national quality criteria for EVT to be eligible for participation in the MR CLEAN-MED [32].

### Randomization and blinding

Patients who are eligible for inclusion in the MR CLEAN-MED will be randomized by the treating physician, before endovascular treatment is started. The randomization procedure is computer- and web-based, using permuted blocks. Back-up assistance by telephone is provided. The allocation sequence has been generated by the independent trial statistician. Randomization is stratified for participating center and in case of participation in MR ASAP for the inclusion in the active treatment arm (nitroglycerine patch group). For each patient that withdraws before the final outcome assessment, an additional patient will be included.

Both patient and treating physician will be aware of the treatment allocation. This open label design was chosen from a safety perspective to avoid potential delay in treatments for serious adverse events (e.g., administer IV protamine sulfate) required for unblinding of the study intervention. Clinical outcomes, such as NIHSS, and serious adverse events are reported by trained research personnel. Trained research personnel unaware of treatment allocation will assess information on outcome at 3 months using standardized forms and procedures during a telephone interview [33, 34]. To guarantee unawareness of the research personnel assessing the outcome at 3 months, they will have no access to the medical records of the patients, instruct patients or relatives before starting the interview not to say anything about the performed procedure or the admission in the hospital, and they will enter the outcome data in a database that is kept separated from the main clinical database. Final assessment of the mRS score at 90 days will be performed by the outcome committee, consisting of trained investigators blinded to the treatment allocation, based on the reports of the telephone interview. Neuroimaging on CT, MRI, and DSA will be assessed by a core laboratory blinded to study treatment allocation. Information concerning treatment allocation will be kept separate from the 90-day follow-up outcome database. The steering committee will be kept unaware of the results of safety assessments and interim analyses. An independent trial statistician will combine data on treatment allocation with the clinical and outcome data to report summaries of trial progress, regular safety assessments, and interim analyses on efficacy and safety to the data safety monitoring board (DSMB).

### Study treatments

The study treatments, IV ASA and/or IV UFH, should be started directly after groin puncture, and in case no rtPA has been administered, prior to EVT. If rtPA administration is not finished at the time of groin puncture, the study treatment should be delayed until the moment that the infusion of the full dose of rtPA is completed. The study treatment should however be started before the EVT procedure has been terminated, i.e., before the catheter has been withdrawn and the entry location has been closed. For both study interventions, IV instead of oral administration was chosen to prevent exclusion of patients with dysphagia and to guarantee fast uptake.

IV ASA will be administered in a single dose of 300 mg. IV UFH will be administered either in a low dose (loading dose of 5000 IU followed by 500 IU/h × 6 h) or a moderate dose (loading dose of 5000 IU followed by 1250 IU/h × 6 h). Study treatments will be combined to increase the efficiency of the trial, under the assumption of independence in mechanisms of action between study treatments, study treatments will be combined. According to the 2 × 3 factorial design, the six possible combinations are the following (Fig. 2): (I) no ASA and no UFH, (II) no ASA and low-dose UFH, (III) no ASA and moderate-dose UFH, (IV) ASA and no UFH, (V) ASA and low-dose UFH, and (VI) ASA and moderate-dose UFH. When the occlusion seen on CTA or MRA is no longer present on first intracranial DSA during EVT before initiation of mechanical treatment (i.e., groin puncture), and the patient has been randomized for UFH, the UFH infusion should be continued for 6 h. In case an untoward event occurs after randomization (e.g., perforation, neurological deterioration), the decision to withhold ASA and/or UFH is left to the discretion of the treating physician. All patients in the study will start or continue non-trial antithrombotic medication for secondary prevention according to local guidelines, which mostly concerns treatment with antiplatelet agents.

### Study procedures

Patients undergo assessment of the NIHSS at baseline, 24 h, and 5–7 days. Certified assessors will carry out the NIHSS assessment. Patients will undergo NCCT and CTA at baseline, as part of usual care. For baseline imaging, MRI and MRA are also permitted. Follow-up imaging can be performed with either NCCT and CTA at 24 h (± 12 h) and NCCT at 5–7 days or discharge, or MRI and MRA at 24 h (± 12 h). If follow-up imaging at 24 h (± 12 h) is performed with MRI, no additional imaging at 5–7 days or discharge is required. The protocol “MRI follow-up investigations” should consist of at least diffusion weighted imaging (DWI), fluid attenuation inversion recovery (FLAIR), T2 weighted image (T2*w), and intracranial three-dimensional time-of-flight (3D-TOF) MRA sequences.

The choice of post-EVT imaging modality (CT or MRI) is left to the individual participating centers, but the chosen modality should be adhered to during the trial in order to prevent confounding by indication. Only in case of contra-indications for MRI, CT imaging may be performed instead and vice versa. The condition of the patient should not drive the decision to deviate from the standard imaging protocol. Follow-up imaging is not part of usual care in every hospital.

Blood samples will be taken from patients when logistics at the participating centers allow this. Blood samples will be drawn at the following time points: (1) within 1 h before groin puncture, (2) within 1 h after EVT, and (3) at 24 h after EVT, if possible during routine blood drawings. We will also take a blood sample if the patient has a regular (not-trial-related) outpatient clinic appointment (2–6 months after treatment). One tube EDTA (± 5 mL), one tube without anticoagulant (± 7 mL), and two tubes citrated blood (2.7 mL) will be drawn every time, which adds up to no more than 20 mL. Substudies may require additional blood tubes, never exceeding 20 mL per drawing. If continuous venous access is available, commonly the case in patients at time point 1, 2, and 3, this will be used. Samples will be stored at − 80 °C for later analysis of procoagulant and genetic factors that may interact with treatment effect. In addition, “waste material” (i.e., retrieved thrombi and blood aspirated during the EVT) will be stored. All biomaterials will be stored for 15 years.

### Deferred consent

**Fig. 4:**
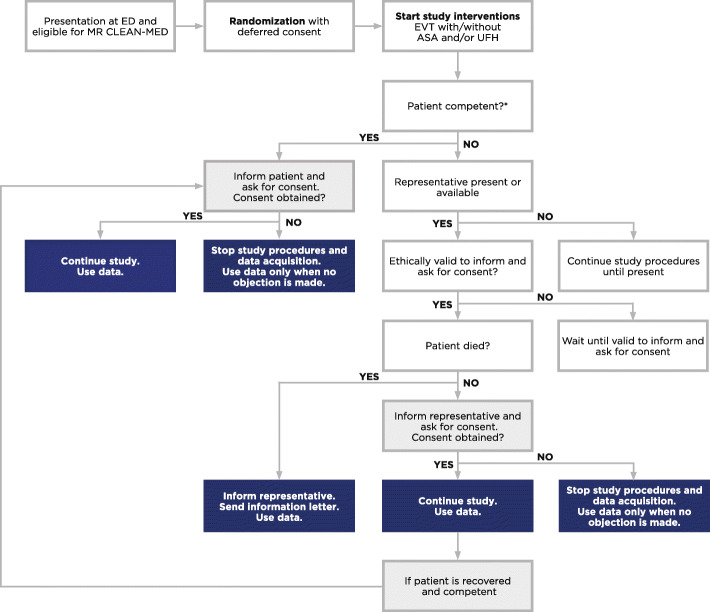
Flow of informed consent procedure in the MR CLEAN-MED. *Abbreviations:* MR CLEAN-MED, multicenter randomized clinical trial of endovascular treatment for acute ischemic stroke. The effect of periprocedural medication: acetylsalicylic acid, unfractionated heparin, both or neither; ED, emergency department; EVT, endovascular treatment; ASA, intravenous acetylsalicylic acid; UFH, intravenous unfractionated heparin. *Captions:* *The patient or representative will be asked to provide consent as early as deemed appropriate and reasonable after hospital admission, ideally before upcoming study procedures after EVT and ultimately before final outcome assessment

MR CLEAN-MED will investigate an acute intervention in an emergency situation concerning a life-threatening disorder. For several ethical and legal reasons, the investigators ask all patients or their representative for written consent after the study treatment(s) and EVT have been carried out (i.e., deferred informed consent). The patient or representative will be asked to provide consent as early as deemed appropriate and reasonable after hospital admission, ideally before upcoming study procedures after EVT and ultimately before final outcome assessment. If a patient or his/her representative refuses to provide consent, participation in the trial will be terminated immediately. Participation in MR CLEAN-MED is voluntary, and the patient or representative may—at any given time—withdraw informed consent without explanation. When consent by proxy has been obtained and the patient recovers, we will again ask for written consent from the patient. If a patient has died before deferred consent was obtained, the representative will be informed about trial participation (Fig. 4).

### Study outcomes

The primary outcome is the score on the modified Rankin Scale at 90 days (± 14 days) [35]. The mRS is the preferred disability parameter of clinical trials in stroke [36]. The mRS is an ordinal hierarchical scale that describes the range of disability encountered post stroke and incorporates six categories from 0 (no symptoms) to 5 (severe disability), and a score of 6 has been added to include “death.” Assessment of outcome on the mRS will be performed by independent assessors, blinded to the allocated and received study treatment.

Secondary outcomes include:

- Recanalization grade (extended Treatment In Cerebral Ischemia [eTICI] score) on final digital subtraction angiography (DSA) after EVT [37];
- Recanalization grade at 24 h (± 12 h), assessed with CTA or TOF-MRA [38];
- Score on the NIHSS at 24 h and 5–7 days, or at discharge [39];
- Follow-up infarct volume, at 5–7 days assessed with NCCT, or at 24 h (± 12 h), assessed with DWI-MRI. Follow-up infarct volume will be assessed with the use of an automated, validated algorithms [40];
- All possible dichotomizations of the mRS at 90 days (± 14 days);
- Score on the EQ-5D-5L and Barthel index at 90 days (± 14 days) [41, 42].

Safety outcomes include:

- Intracerebral hemorrhage according to the Heidelberg Bleeding Classification [43];
- SICH scored according to the Heidelberg Bleeding Classification (with the addition of sICH that led to death and that was identified as the predominant cause of the neurologic deterioration) [43];
- Extracranial hemorrhage requiring transfusion or resulting in death;
- Embolization in new territory on DSA during EVT;
- Infarction in new territory within 5–7 days assessed with NCCT or 24 h (± 12 h) assessed with DWI-MRI;
- Death from all causes within 90 days

All imaging-related outcomes on CT, MRI, and DSA will be assessed by an independent core laboratory blinded to study treatment allocation. Clinical outcomes such as NIHSS and serious adverse events are reported by trained research personnel.

### (Serious) adverse event reporting

Safety is an issue of concern as both ASA and UFH could increase bleeding risk. Adverse events are defined as any undesirable experience occurring to a subject during the study, whether or not it is considered related to the investigational product. All adverse events reported spontaneously by the subject or observed by the investigator or his/her staff will be recorded. A serious adverse event is any untoward medical occurrence or effect that (I) results in death, (II) is life threatening (at the time of the event), (III) requires hospitalization or prolongation of existing inpatients’ hospitalization, or (IV) results in persistent or significant disability or incapacity. The (local) investigator will report the following SAEs occurring in the study period to the sponsor without undue delay of obtaining knowledge of the events: death from any cause, sICH defined according to the Heidelberg criteria, extracranial hemorrhage, cardiac ischemia, pneumonia, allergic reactions, and new ischemic stroke in a different vascular territory. Events that result in any of the outcomes listed, according to appropriate medical judgment, if no medical or surgical intervention would have been carried out, will also be considered a serious adverse event. Serious adverse events that meet the aforementioned criteria will be reported to the sponsor, within 24 h after coming to notice of the (local) investigator, by making use of the appropriate forms in the eCRF, which will automatically lead to notification of the study coordinator. Elective hospital admission will not be considered a serious adverse event. Technical complications or vascular damage at the target lesion such as perforation or dissection that do not lead to clinically detectable SAEs, and neurological deterioration not caused by intracranial hemorrhage or new ischemic stroke, are considered as consistent with the natural course of the ischemic stroke and should be reported at the patient’s 90 days follow-up.

### Safety registry

Due to the deferred consent procedure, the study treatment will have been administered to patients prior to obtaining informed consent. The procedure requires that all information on patients who did not provide consent after EVT is discarded and deleted. This may be against the interest of patients who did provide consent, and against the interest of the general public, as patients with sICH and other serious adverse events might be more likely to refuse consent for participation. Not considering these records might very well result in an underestimation of the true safety and validity of the data, and it might lead to undetected safety concerns for all consenting patients in the trial. To overcome this concern, we will register the following variables in a strictly anonymized safety registry for all patients, irrespective of whether a patient has provided written informed consent: patient’s study number, study treatment, in-hospital sICH occurrence (*yes/no*), and in-hospital survival status (*yes/no*). All other information will be completely erased from the patient’s study record in case no consent is provided. The link to the study database will be erased from the patient’s medical record.

### Data and Safety Monitoring Board

The independent Data and Safety Monitoring Board (DSMB) consists of a neurologist, a neuro-interventionist, and an independent statistician. The DSMB will meet at least annually or after inclusion of each 300 patients (whichever comes first) to monitor the efficacy and safety of the study treatments. The DSMB will evaluate the occurrence of unwanted effects by study treatments and by center. During the period of patient enrollment, short safety reports are made by the independent statistician of the trial after the occurrence of every 5 sICHs or after the occurrence of every 10 deaths, whichever comes first. Depending on the results of previous analyses, the DSMB may propose to the steering committee to relax the criterion of 5 sICH or 10 deaths. Safety of the study treatments in terms of sICH risk and all-cause mortality will be evaluated based on the safety registry. Also, during the period of patient enrollment, interim analyses on major endpoints (including serious adverse events believed to be due to treatment) will be supplied by the independent statistician of the trial (annually or as soon as possible after inclusion of 300 patients, whichever comes first), in strict confidence, to the chairman of the DSMB along with any other analyses that the committee may request. In the light of the safety reports and interim analyses, the DSMB will advise the chairman of the steering committee if, in their view, the randomized comparisons in the trial have provided both (1) “proof beyond reasonable doubt” that for all, or for some specific types of patients, one particular treatment is clearly indicated or clearly contraindicated in terms of a net difference in outcome, and (2) evidence that might reasonably be expected to materially influence patient management. Appropriate criteria of proof beyond reasonable doubt cannot be prespecified precisely, but a difference of at least 3 standard deviations in an interim analysis of a major endpoint may be needed to justify halting or modifying the study prematurely. This criterion has the practical advantage that the number of interim analyses is of little importance. The principal investigators (PIs), study personnel, and steering committee will remain blinded to the treatment allocation in the dataset and to the results of the safety assessments and interim analyses.

### Sample size

Power was estimated by simulation [44]. For the control arm in the study, the distribution over the 7-point mRS was based on data of the MR CLEAN trial: mRS 0, 3%; mRS 1, 9%; mRS 2, 21%; mRS 3, 18%; mRS 4, 22%; mRS 5, 6%; and mRS 6, 21%. For both ASA and UFH, we assume a favorable effect with a common odds ratio of 1.27, which corresponds to an absolute risk difference of having a score on the mRS of 0–2 of approximately 5%. Covariate adjustment will be used, which reduces the required sample size by approximately 25% [45, 46]. We aim to include 1500 patients, which will provide 84% power to detect a true difference in outcome between the ASA and the control arm, and 78% power to detect a true difference in outcome between any of the low-dose UFH, moderate-dose UFH, and control arm (two-sided alpha = 0.05). No adjustments for multiple comparisons will be made.

### Statistical analyses

The analysis and reporting of the trial will be in accordance with the CONSORT guidelines [47].

The main treatment contrasts that will be analyzed are:

- ASA vs. no ASA
- Any dose UFH vs. no UFH

In addition, as secondary analysis, we will compare:

- Low-dose UFH vs. no UFH
- Moderate-dose UFH vs. no UFH
- Low-dose UFH vs. moderate-dose UFH

All analyses will be performed according to the intention-to-treat principle. Baseline data by treatment allocation will be reported with standard statistical procedures, and missing values for baseline characteristics will be reported. Missing baseline characteristics will be imputed using multiple regression imputation. The primary effect estimate, which is the common odds ratio for a shift on the 7-category mRS at 90 days, will be assessed by means of an ordinal logistic regression analysis. Secondary effect estimates will be assessed by means of linear, logistic, or ordinal logistic regression analyses, as appropriate. Pre-specified adjustments will be made for known prognostic variables including: age, time from onset to door of EVT center, time from door EVT center to groin puncture, baseline NIHSS, pre-stroke mRS, and collateral score. Adjusted and unadjusted estimates will be reported as a beta, odds ratio, or common odds ratio with their 95% confidence intervals. We assume the effects of ASA and UFH to be independent, but this assumption will be tested with a test for interaction. Before follow-up of all included patients is completed, a statistical analysis plan will be developed and published that specifies the hypotheses to be tested and the more detailed statistical methods to analyze treatment effects on secondary outcomes, adjustments for covariates and subgroup analyses. We will interpret the common odds ratio as the best estimate of an average treatment effect, and therefore, no formal testing of the proportionality assumption is necessary.

### Data management

All MR CLEAN-MED data are entered into a web-based trial management system that allows for edit and audit trails, by trained local research nurses. Case report forms can be found on the website (http://www.mrcleanmed.nl/). Patient records are coded by a unique study number. The local investigators will keep a list showing codes and names. Unique documents with identifying information will be stored separately from the study database in digital files, categorized by study number on a secure drive system, only accessible to the study coordinators. Data will be monitored for completeness, consistency, and validity by the study coordinators through automated data checks. Twenty-five percent of local data are carefully reviewed against source data, based on a pre-assessed risk evaluation and in accordance with Dutch standards, by an independent monitor performing two to three visits per year during the study period (Additional file 1). The database will be closed within 1 month after the last scheduled follow-up date of the last included patient.

### Study organization

**Fig. 5:**
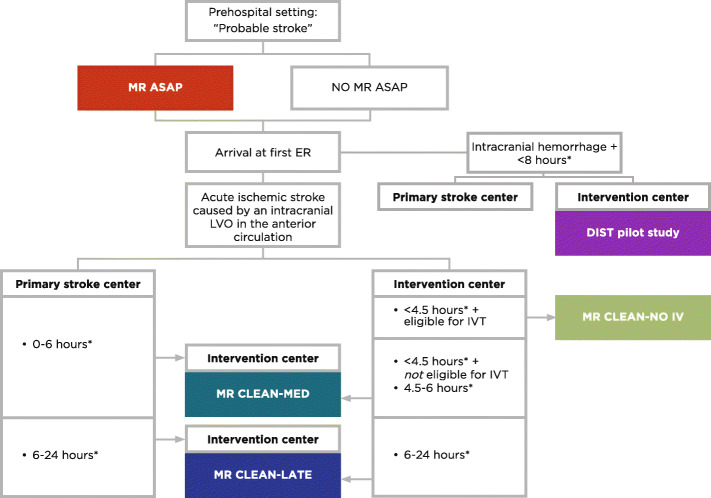
Flow of patients in the acute stroke trials of the Collaboration for New Treatments of Acute Stroke (CONTRAST) consortium. *Abbreviations: *MR ASAP, Multicentre Randomised trial of Acute Stroke treatment in the Ambulance with a nitroglycerin Patch; ED, emergency department; DIST pilot study, Dutch Intracerebral Hemorrhage Surgery Trial - pilot study; minimally-invasive endoscopy-guided surgery for spontaneous intracerebral hemorrhage; LVO, large vessel occlusion; IVT, intravenous thrombolysis with alteplase; MR CLEAN-MED, multicenter randomized clinical trial of endovascular treatment for acute ischemic stroke. The effect of periprocedural medication: acetylsalicylic acid, unfractionated heparin, both or neither; MR CLEAN-NO IV, intravenous treatment followed by endovascular treatment versus direct endovascular treatment for acute ischemic stroke caused by a proximal intracranial occlusion; MR CLEAN-LATE: Multicenter Randomized Clinical Trial of Endovascular Stroke treatment in The Netherlands for Late arrivals. *Captions:* *Considerations: The CONTRAST trials are independent clinical trials. Patients included in MR ASAP may also be included in one of the other trials. We will perform pre-specified subgroup analyses to test for interaction between the different study treatments. At the first ED (i.e., primary stroke center or participating EVT center), all patients with a probable diagnosis of acute stroke will undergo non-invasive imaging to differentiate between cerebral infarction or intracranial hemorrhage and to assess an intracranial LVO in the anterior circulation. When the first ED is a primary stroke center and the patient could be eligible for DIST pilot study, MR CLEAN-MED, or MR CLEAN-LATE, the patient should be transferred to a participating EVT center. Patients arriving at a primary stroke center first will generally not be eligible for MR CLEAN-NO IV, since IVT cannot be withheld until after patient transfer to the EVT center, unless the perceived contraindications for IVT are not present anymore upon arrival at the EVT center. Then, inclusion in MR CLEAN-NO IV will have priority over inclusion in other trials. Competition between the three MR CLEAN trials will not occur

MR CLEAN-MED is embedded in the Collaboration for New Treatments of Acute Stroke (CONTRAST) consortium, a nationwide collaboration of clinical and translational scientists. The CONTRAST consortium will perform five large RCTs in stroke patients to test novel treatment strategies, aimed at preservation of ischemic tissue and improving outcome after stroke (Multicentre Randomised trial of Acute stroke treatment in the Ambulance with a nitroglycerin Patch [MR ASAP, ISRCTN99503308] [31]; intravenous treatment followed by endovascular treatment versus direct endovascular treatment for acute ischemic stroke caused by a proximal intracranial occlusion [MR CLEAN-NO IV, ISRCTN80619088]; the current study: Multicenter randomized clinical trial of endovascular treatment for acute ischemic stroke. The effect of periprocedural medication: acetylsalicylic acid, unfractionated heparin, both or neither [MR CLEAN-MED, ISRCTN76741621]; Multicenter Randomized Clinical Trial of Endovascular Treatment of Acute Ischemic Stroke in The Netherlands for Late arrivals [MR CLEAN-LATE, ISRCTN19922220]; The Dutch ICH Surgery Trial - pilot study; minimally-invasive endoscopy-guided surgery for spontaneous intracerebral hemorrhage [DIST, NTR7180]. Although MR CLEAN-NO IV, MR CLEAN-MED, and MR CLEAN-LATE, which all aim to improve outcome after EVT by focusing on the optimization of EVT and the expansion of its indication, draw from the same pool of patients with acute ischemic stroke, there is no competition between the three trials (Fig. 5). All studies are independent clinical trials, but investigators collaborate closely and the trials share the same data structure and format, imaging and clinical assessment procedures, and outcome, imaging, and SAE assessment committees. Patients enrolled in MR CLEAN-NO IV, MR CLEAN-MED, or MR CLEAN-LATE can also participate in MR ASAP, for which patients will be stratified.

The MR CLEAN-MED is guided by several MR CLEAN-MED-organized and CONTRAST-organized committees:

*The steering committee* of the trial consists of all local PIs of the participating centers. Each participating center has two local PIs: a vascular neurologist and a neuro-interventionist. The steering committee will meet at least annually. Final decisions concerning protocol changes, publication, and reporting will be made by the steering committee. The steering committee is chaired by the central PIs of the trial. Decisions will be made in consensus, but if unavoidable by majority vote. Day to day conduct of the trial will be managed by the trial coordinators, who will be supervised by the central PIs of the trial.

*The executive committee* of the trial consists of the central PIs of the trial, a representation of local PIs, including the PIs of the two other MR CLEAN II trials, and of the study coordinators. They meet regularly, discuss trial progress, and prepare information for the steering committee.

*The writing committee* consists of the executive committee and local PIs of the five collaborating centers that have contributed the most patients to the trial in the first 2 years of trial execution. The task of the writing committee is to prepare the main publication which will be drafted by the study coordinators, supervised by the two central PIs. Typically, the main paper will be authored by the study coordinators, the local PIs, the committee members, the central PIs, the coordinators of the two other MR CLEAN trials, and data management group, in name of all MR CLEAN-MED investigators. Authorship has to comply with the criteria of the International Committee of Medical Journal Editors (IMCJE at http://www.icmje.org/) [48].

The other trial committees are not trial specific and will be formed in collaboration with the four CONTRAST randomized clinical trials on acute stroke: MR ASAP, MR CLEAN-LATE, MR CLEAN-MED, and MR CLEAN-NO IV. These are the imaging committee, the adverse event committee, and the outcome committee. The committees will regularly report to the steering committees of the involved trials.

*The imaging committee* is chaired by the CONTRAST imaging work package leaders (CM and AL) and consists of neuroradiologists from the collaborating centers. Their task is to assess and evaluate masked baseline and follow-up imaging, which is performed per protocol and stored in a central web-based database (XNAT, https://www.xnat.org/). Assessments will be stored in research forms and entered in the clinical database, which will be accessible to investigators after approval by the Steering committee.

*The adverse event committee* consists of at least 3 members, including a neurologist and a neuroradiologist. Their task is to oversee and review all reported serious adverse events.

*The outcome committee* consists of at least 3 members, all seasoned neurologists. Their task is to evaluate all coded and masked structured reports of the outcome assessments at 90 days of patients in the trials. This way, we can ensure blind outcome assessment.

The investigators and collaborators of MR CLEAN-MED are listed in the Appendix.

Strategies for improving adherence to the intervention protocol and other study procedures and for achieving adequate participant enrollment include training sessions at all participating centers, regular newsletters and research meetings with all collaborators, and monthly telephone meetings with the study coordinators and central PIs of the MR ASAP, MR CLEAN-NO IV, MR CLEAN-MED, and MR CLEAN-LATE.

### Ethical considerations

The MR CLEAN-MED protocol, including the template informed consent forms, which can be found on http://www.mrcleanmed.nl/ has been approved for the Netherlands by the central medical ethics committee and research board of the Erasmus MC University Medical Center, Rotterdam, the Netherlands (MEC-2017-366) before the start of the trial. The study will be conducted according to the principles of the Declaration of Helsinki (7th revision, October 2013), ICH-GCP, the Dutch Medical Research Involving Human Subjects Act (WMO) and when it becomes applicable in accordance with regulations of other countries with participating centers. The current manuscript is based on protocol version 1.6 (April 2019). The most up-to-date approved trial protocol including protocol version and amendments can be found on the website (http://www.mrcleanmed.nl/).

#### Trial status

**Fig. 6:**
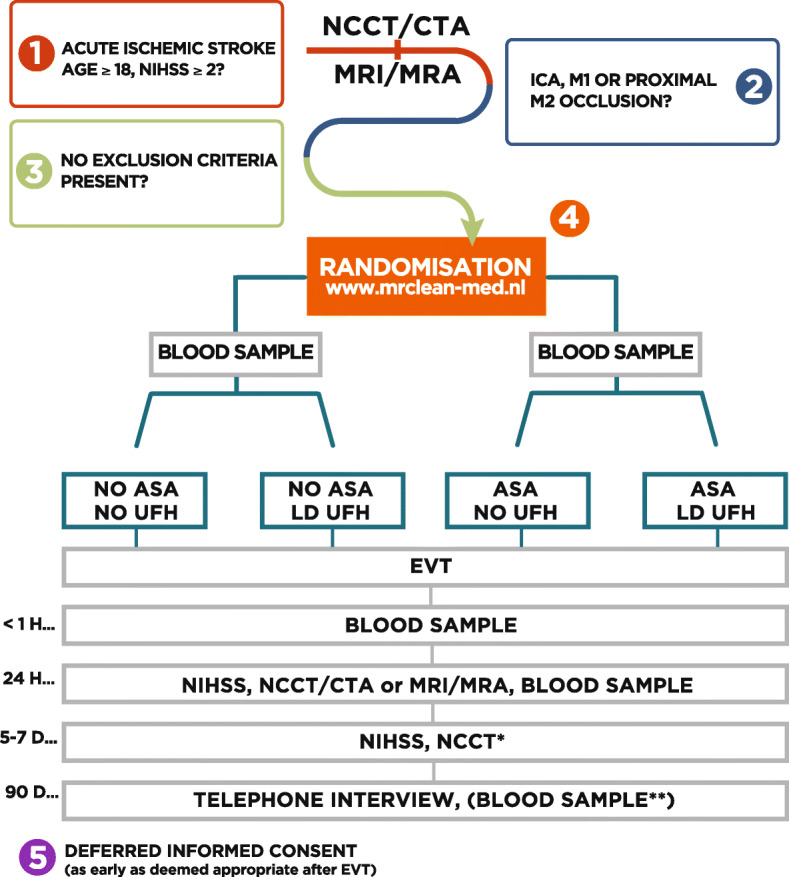
Flow of patients in the MR CLEAN-MED—modification after the recommendation of the DSMB to stop recruiting patients for moderate-dose unfractionated heparin. *Abbreviations: *ASA, intravenous acetylsalicylic acid; CTA, computed tomography angiogram; UFH, intravenous unfractionated heparin; EVT, endovascular treatment; LD, low dose; MD, moderate dose; MRI, magnetic resonance imaging; MRA, magnetic resonance angiography; NCCT, non-contrast computed tomography; NIHSS, National Institutes of Health Stroke Scale. Captions: *Only to be performed if imaging at 24 hour was acquired with CT; **Blood sample drawn only in case of regular outpatient clinic appointment within 2-6 months after intervention

The initial approval of the MR CLEAN-MED trial protocol by the ethical board covered approval of patient enrollment in the four organizing centers of four stroke trials performed by the CONTRAST collaboration. For logistic reasons, approval was given for 16 other centers at a later stage. The first patient was enrolled in the MR CLEAN-MED in January 2018. The DSMB did not report any safety concerns following the first three safety assessments. However, after receipt of the 4th safety report on April 16, 2019, the DSMB recommended unanimously that the steering committee should consider stopping the moderate-dose UFH arm of the trial, but should continue the other arms of the trial. The grounds for stopping this specific arm were related to safety rather than efficacy. The steering committee of the trial has acted upon receiving this advice and directly stopped inclusion in the moderate-dose UFH arms of the trial. At the time point of receiving the DSMB’s advise, 137 patients were enrolled in the trial, of which 46 patients in the moderate-dose UFH arm. No patients have been included in the moderate-dose UFH arm after receipt of the DSMB recommendation. After consulting the medical ethics committee, the inclusion of patients in the ASA and low-dose UFH arms was continued the next day (Fig. 6). Meanwhile, patients, family, and representatives were contacted personally and regulatory bodies were notified. In the 4th safety report, the DSMB advised to further evaluate the efficacy and safety of the moderate-dose UFH arm, which will be compared to the blinded data of the other arms. Results from this analysis have been reported on scientific meetings and will be submitted for publication [49, 50]. On May 31, 2020, a total of 5 Dutch and 5 French sites agreed to participate in MR CLEAN MED, and 441 patients were included in the trial by 13 enrolling centers. Recruitment is expected to be completed by the end of 2021. More information about the MR CLEAN-MED, including progress of the trial and participating centers can be found on the website (http://www.mrcleanmed.nl/).

## Discussion

MR CLEAN-MED—a multicenter RCT investigating the effect of periprocedural ASA and UFH, alone or in combination, in patients with acute ischemic stroke who undergo EVT within 6 h after symptom onset for a confirmed intracranial large vessel occlusion in the anterior circulation—is being conducted in the framework of the CONTRAST consortium (https://www.contrast-consortium.nl/) in continuation of the MR CLEAN trial [51] and the MR CLEAN-Registry [52] to further improve outcomes for patients who undergo EVT. This trial will provide evidence whether adjunctive periprocedural therapy with ASA and/or UFH leads to improved microvascular reperfusion and better outcomes despite a possibly increased risk of sICH in these patients.

### Other ongoing trials

Next to the MR CLEAN-MED, there are currently no other ongoing trials investigating the effect of ASA and/or UFH in patients with acute ischemic stroke who will undergo EVT.

### Expected benefit

The trial design is pragmatic and the trial results should be generalizable and representative of clinical practice. We chose to include the most unselected patient population—in which EVT was proven effective—in the MR CLEAN-MED, so that the study treatments may be extrapolated to the broadest and most diverse patient group, if proven effective.

This implies that both ASA and UFH as an adjunctive treatment to EVT may be given to a broad selection of patients undergoing EVT. We consider both ASA and UFH suitable for evaluation with a phase 3 RCT, as both study treatments are well known and have been used for similar endovascular procedures in the fields of neurology and cardiology for several decades. There is extensive clinical experience with the use of both agents, they are both widely accessible, cheap, easy to administer, and for UFH the activity is easily reversed—also in adjunction to EVT. Therefore, if a significant treatment effect of ASA and/or UFH will be proven in the MR CLEAN-MED, both treatments could also be easily implemented in clinical practice on a large scale. Considering the low costs of this medication, we expect the treatment to be cost-effective as well. Consequently, a substantial number of patients could potentially profit from these treatments.

### Limitations and concerns

The underlying assumption of independence of study treatment effect on the primary outcome in a randomized trial with a factorial design is important, because it allows analysis of treatment effects separately for both treatments. We consider the assumption reasonable for the main effects on functional outcome, as the underlying mechanisms differ (inhibition of platelet activation versus interference with coagulation factors thrombin, factor Xa, other proteases through activation of antithrombin and by degrading NETs). However, we will analyze whether the assumption of independence of the effect of aspirin and heparin on the occurrence of sICH is true. If the assumption of independence is violated, we will analyze stratified treatment effects.

Inherently to the acute setting of the trial and necessity for deferral of consent, bias could have been introduced by selective patient refusal (e.g., in case of a poor clinical condition). The trial will provide generalizable results regarding safety of the study treatments, as all randomized patients will be registered in the safety registry providing information on in-hospital sICH risk and mortality. It is not possible to register mRS scores of randomized patients who refused to provide consent out of respect of each patient’s autonomy. Therefore, the estimate of the treatment effect on the primary outcome should be interpreted in the light of the refusal rate. As we expect the refusal rate to be low, we anticipate that the impact on the generalizability of the trial’s results will be low.

Based on the available literature, upon start of the trial, we considered the risk of sICH associated with the administration of both UFH doses and/or ASA acceptable in the light of expected improved functional outcomes. This was also reflected by the systematic use of UFH for this indication in a substantial number of centers in the Netherlands and in other countries [28–30]. Moreover, experimental work showed that the immediate use (< 6 h) of ASA and/or UFH could add to improvement of outcomes by preventing or limiting microvascular occlusion within the regions of ischemic injury [5, 53, 54]. The risk of sICH risk remains an important concern in the MR CLEAN-MED. We monitor the occurrence of sICH strictly by performing regular safety assessments in consultation with the DSMB. We also evaluate the safety of the study medication in terms of sICH rate and all-cause mortality in the anonymized safety registry, which includes all treated patients.

There is no clear evidence which UFH dose and regimen may be most effective and safe. We had chosen to investigate the efficacy of two different UFH doses to evaluate a possible dose effect. The protocol for the different dosages of UFH are based on findings from the PROACT I and II and IST trials and on experience in later thrombectomy studies [28–30, 55, 56]. The PROACT I trial compared intra-arterial recombinant pro-urokinase in combination with IV UFH to IV UFH alone for patients with a visible middle cerebral artery occlusion. The bleeding risk in the UFH alone arm of 7.1% seemed acceptable for both the high dose (100 IU/kg bolus followed by 1000 IU/h continuous infusion for 4 h) and low-dose arms (dose 2000 IU bolus followed by 500 IU/h continuous infusion for 4 h). In the high-dose UFH group of the International Stroke Trial (IST), in which patients received 12,500 IU UFH subcutaneous twice daily up to 14 days, the absolute risk of sICH was low (2.0%) [56]. These results suggested that the low and moderate doses of UFH in MR CLEAN-MED would both be associated with acceptable risks. Nevertheless, the DSMB recommended the steering committee to stop enrollment in the moderate-dose UFH arm of the trial based on safety concerns. Therefore, this arm has been removed from the trial and safety and outcome results will be reported separately, without compromising the blinding of investigators to results of the other trial arms. As inclusion in the moderate-dose UFH arm of the trial has been permanently discontinued, we will now only investigate treatment effects for ASA and for low-dose UFH.

The steering committee of the MR CLEAN-MED advised per July 19, 2019, for reasons of homogeneity among centers to limit intra-arterial flushing of the sheath with UFH during EVT up to 2500 IU per liter.

### Deferral of consent

In MR CLEAN-MED, we use a deferred consent procedure. The primary reason for this approach is that in ischemic stroke, acute treatments are based on the “time is brain” principle, in order to reduce loss of brain tissue as time progresses. In patients treated with EVT, each hour delay to reperfusion is associated with an increase in absolute risk of disability of 6–7% [1]. First of all, experience in MR CLEAN indicates that a proper informed consent procedure takes more than 1 h, even when a legal representative is involved. This would lead to an unacceptable delay, considering the time-dependent effect of EVT. Second, most patients with acute neurological deficits (such as impaired consciousness or aphasia) are not capable of decision making before enrollment in a trial. In the MR CLEAN Registry, 80 to 96% of the acute ischemic stroke patients eligible for EVT were in retrospect considered to lack decision-making capacity at admission, based on neurological symptoms potentially interfering with their capacity to decide about trial participation [57]. Exclusion of these patients might lead to selection bias and reduced generalizability of the trial results. Lastly, the decision-making capacity for trial participation in an emergency situation is also reduced by stress and by the complexity and volume of the provided information. Thus, the use of the deferred consent procedure is likely to increase patient enrollment and to reduce selection bias. However, if a substantial number of patients or representatives object to enrollment after EVT, this could actually contribute to a different kind of selection bias, particularly if this disproportionally concerns patients with adverse events and poor clinical outcome. Postponing consent seems tolerated by patients and their relatives in several clinical studies and trials [58–65]. However, a substudy of the ESCAPE trial (the Endovascular Treatment for Small Core and Anterior Circulation Proximal cOcclusion with Emphasis on Minimizing CT to Recanalization Times) showed that the majority of patients or their representatives disagreed with the use of deferred consent [66]. Yet, none of the patients enrolled with deferred consent in this trial withdrew consent later, and patients agreed with the conditions used to justify deferred consent procedures. A separate substudy within the CONTRAST consortium, in the form of a survey, will be carried out to further elucidate the acceptability of the deferred consent procedure in acute stroke trials.

## Conclusion

MR CLEAN-MED is a pragmatic randomized clinical trial with a PROBE design. ASA and UFH are well known and available everywhere. When one or both of the study treatments show the anticipated effect on outcome, we will be able to improve outcome of patients treated with EVT by 5%. This amounts to more than 50 patients annually in the Netherlands, more than 1800 in Europe, and more than 1300 each year in the USA [67, 68].

### Supplementary information

**Additional file 1.** MR CLEAN-MED monitoring plan (Dutch).

## Data Availability

When the database is closed, all CONTRAST investigators will have access to the data. Data will be made available for replication of the study results upon reasonable request to the principal investigators, 18 months after publication of the first paper. Data may also be shared with non-commercial parties for scientific purposes, including individual patient meta-analyses, and with commercial parties for FDA approval. Consent will be asked specifically for these purposes.

## Appendix

### MR CLEAN-MED investigator list

#### MR CLEAN-MED:

Principal investigators:

Diederik Dippel (MD, PhD),^1^ Aad van der Lugt (MD, PhD)^1^

Study coordinators:

Bob Roozenbeek (MD, PhD),^1^ Vicky Chalos (MD) ^1^, Rob van de Graaf (MD),^1^ Wouter van der Steen (MD)^1^

Local principal investigators

Bob Roozenbeek (MD, PhD),^1^ Adriaan van Es (MD, PhD),^1^ Jonathan Coutinho (MD, PhD),^2^ Bart Emmer (MD, PhD),^2^ Inger de Ridder (MD, PhD),^4^ Wim van Zwam (MD, PhD),^4^ Bart van der Worp (MD, PhD),^5^ Rob Lo (MD, PhD),^5^ Koos Keizer (MD, PhD),^6^ Rob Gons (MD),^6^ Lonneke Yo (MD, PhD),^6^ Jelis Boiten (MD, PhD),^7^ Ido van den Wijngaard (MD, PhD),^7^ Jeanette Hofmeijer (MD, PhD),^8^ Jasper Martens (MD),^8^ Wouter Schonewille (MD, PhD),^9^ Jan Albert Vos, (MD, PhD),^9^ Anil M. Tuladhar (MD, PhD),^10^ Sjoerd Jenniskens (MD, PhD),^10^ Karlijn de Laat (MD, PhD),^11^ Lukas van Dijk (MD, PhD),^11^ Heleen den Hertog (MD, PhD),^12^ Boudewijn van Hasselt (MD),^12^ Paul Brouwers (MD, PhD),^13^ Emiel Sturm (MD),^13^ Michel Remmers (MD),^14^ Thijs de Jong (MD),^14^ Anouk Rozeman (MD),^15^ Otto Elgersma (MD, PhD),^15^ Maarten Uyttenboogaart (MD, PhD),^16^ Reinoud P.H. Bokkers (MD, PhD),^16^ Julia van Tuijl (MD),^17^ Issam Boukrab (MD)^17^

Local trial collaborators:

Executive and writing committee:

Diederik Dippel (MD, PhD),^1^ Aad van der Lugt (MD, PhD),^1^ Adriaan van Es (MD, PhD),^1^ Heleen den Hertog (MD, PhD),^13^ Julie Staals (MD, PhD),^4^ Lukas van Dijk (MD, PhD),^11^ Sjoerd Jenniskens (MD, PhD),^10^ Yvo Roos (MD, PhD),^2^ Charles Majoie (MD, PhD),^2^ Robert van Oostenbrugge (MD, PhD),^4^ Wim van Zwam (MD, PhD),^4^ Rob van de Graaf (MD),^1^ Wouter van der Steen (MD),^1^ Bob Roozenbeek (MD, PhD)^1^

Data Safety Monitoring Board:

Peter Rothwell (MD, PhD) *– Chair,*^*20*^ Andrew Molyneux (MD, PhD)^20^, Joanna Moschandreas (MD, PhD)^20^

Independent trial statistician:

Daan Nieboer (MSc)^1^

Advisory Board:

Gregory del Zoppo (MD, PhD)^21^

CONTRAST clinical trial collaborators:

Research leaders:

Diederik Dippel (MD, PhD),^1^ Charles Majoie (MD, PhD)^2^

Consortium coordinator:

Rick van Nuland, PhD (Lygature)^3^

Imaging assessment committee:

Charles Majoie (MD, PhD) *– Chair*,^2^ Aad van der Lugt (MD, PhD) *– Chair*,^1^ Wim van Zwam (MD, PhD),^4^ Alida Annechien Postma (MD, PhD),^22^ René van den Berg, (MD, PhD),^2^ Ludo Beenen (MD),^2^ Bart Emmer (MD, PhD),^2^ Adriaan van Es, (MD, PhD),^1^ Pieter-Jan van Doormaal (MD),^1^ Geert Lycklama (MD, PhD),^7^ Ido van den Wijngaard (MD, PhD),^18^ Albert Yoo (MD, PhD),^19^ Lonneke Yo (MD, PhD),^6^ Jasper Martens (MD),^8^ Sebastiaan Hammer (MD, PhD)^11^, Stefan Roosendaal (MD, PhD),^2^ Anton Meijer (MD, PhD),^10^ Menno Krietemeijer (MD)^6^, Reinoud P.H. Bokkers (MD, PhD)^16^, Anouk van der Hoorn (MD, PhD)^16^, Dick Gerrits (MD)^13^

Adverse event committee:

Robert van Oostenbrugge (MD, PhD) *– Chair*,^4^ Bart Emmer (MD, PhD),^2^ Jonathan Coutinho (MD, PhD),^2^ Ben Jansen (MD)^17^

Outcome assessment committee:

Yvo Roos (MD, PhD) *– Chair*,^2^ Sanne Manschot (MD, PhD),^7^ Diederik Dippel (MD, PhD),^1^ Henk Kerkhoff (MD, PhD),^15^ Ido van den Wijngaard (MD, PhD),^18^ Jonathan Coutinho (MD, PhD),^2^ Peter Koudstaal (MD, PhD),^1^ Koos Keizer (MD, PhD),^6^ Jelis Boiten (MD, PhD)^7^

Data management group:

Hester Lingsma (PhD),^1^ Diederik Dippel (MD, PhD)^1^, Vicky Chalos (MD),^1^ Olvert Berkhemer (MD, PhD)^1^

Imaging data management group:

Aad van der Lugt (MD, PhD), Charles Majoie (MD, PhD), Adriaan Versteeg^1^, Lennard Wolff (MD),^1^ Jiahang Su (MSc)^1^

Biomaterials and translational group:

Hugo ten Cate (MD, PhD),^4^ Moniek de Maat (PhD)^1^, Samantha Donkel (MD),^1^ Heleen van Beusekom (PhD),^1^ Aladdin Taha (MD)^1^

Local collaborators:

Vicky Chalos (MD),^1^ Kilian Treurniet (MD),^2^ Sophie van den Berg (MD),^2^ Natalie LeCouffe (MD),^2^ Rob van de Graaf (MD),^1^ Robert-Jan Goldhoorn (MD),^4^ Aladdin Taha (MD),^1^ Samantha Donkel (MD),^1^ Wouter Hinsenveld (MD),^4^ Anne Pirson (MD),^4^ Lotte Sondag (MD),^10^ Manon Kappelhof (MD),^2^ Rik Reinink (MD),^5^ Manon Tolhuisen (MD),^2^ Josje Brouwer (MD),^2^ Lennard Wolff (MD),^1^ Sabine Collette,^16^ Wouter van der Steen (MD),^1^ Simone Uniken Venema (MD),^5^ Susan G.H. Olthuis (MD),^4^ Floor M.E. Pinkaers (MD),^4^

Research nurses:

Martin Sterrenberg,^1^ Naziha El Ghannouti,^1^ Sabrina Verheesen,^4^ Rita Sprengers,^2^ Wilma Pellikaan,^9^ Yvonne Drabbe,^11^ Joke de Meris,^7^ Michelle Simons,^8^ Hester Bongenaar,^6^ Anja van Loon,^14^ Eva Ponjee,^12^ Rieke Eilander,^12^ Suze Kooij (BSc),^15^ Marieke de Jong,^16^ Esther Santegoets,^17^ Friedus van der Minne^16^

Study monitors:

Leontien Heiligers^1^, Yvonne Martens^1^

Affiliations

^1^ Erasmus MC University Medical Center, Rotterdam, the Netherlands;

^2^ Amsterdam UMC, location AMC, University of Amsterdam, Amsterdam, the Netherlands;

^3^ Lygature, Utrecht, the Netherlands;

^4^Cardiovascular Research Institute Maastricht (CARIM), Maastricht University Medical Center, Maastricht, The Netherlands;

^5^ University Medical Center Utrecht, Brain Center, Utrecht, the Netherlands;

^6^ Catharina Hospital, Eindhoven, the Netherlands;

^7^Haaglanden Medical Center, the Hague, the Netherlands;

^8^ Rijnstate Hospital, Arnhem, the Netherlands;

^9^ St. Antonius Hospital, Nieuwegein, the Netherlands;

^10^ Radboud University Medical Center, Nijmegen, the Netherlands;

^11^ HagaZiekenhuis, the Hague, the Netherlands;

^12^ Isala Klinieken, Zwolle, the Netherlands;

^13^Medisch Spectrum Twente, Enschede, the Netherlands;

^14^ Amphia Hospital, Breda, the Netherlands;

^15^Albert Schweitzer Hospital, Dordrecht, the Netherlands;

^16^ University Medical Center Groningen, University of Groningen, the Netherlands;

^17^Elisabeth-TweeSteden Hospital, Tilburg, the Netherlands;

^18^ Leiden University Medical Center, the Netherlands;

^19^ Texas Stroke Institute, Plano, Texas, United States of America

^20^ John Radcliffe Hospital, Oxford, United Kingdom)

^21^University of Washington, Seattle, Washington, United States

^22^ School for Mental Health and Sciences (Mhens), Maastricht University Medical Center, Maastricht, The Netherlands

## Abbreviations

ASA: Acetylsalicylic acid
NCCT: Non-contrast computed tomography
CTA: Computed tomography angiography
DSA: Digital subtraction angiography
DSMB: Data safety monitoring board
EVT: Endovascular treatment
EQ-5D-5L: A standardized instrument developed by the EuroQol Group as a measure of health related quality of life, which comprises 5 dimensions with each 5 levels of severity
IMR: Incomplete microvascular reperfusion
IU: International standard unit
IV: Intravenous
MRI: Magnetic resonance imaging
MRA: Magnetic resonance angiography
mRS: Modified Rankin Scale
NET: Neutrophil extracellular traps
NIHSS: National Institutes of Health Stroke Scale
PI: Principal investigator
PROBE: Prospective randomized open blinded end-point
RCT: Randomized controlled trial
rtPA: Recombinant tissue plasminogen activator
(S)AE: (Serious) adverse event
sICH: Symptomatic intracranial hemorrhage
UFH: Unfractionated heparin
WMO: Medical Research Involving Human Subjects Act (in Dutch: Wet Medisch-wetenschappelijk Onderzoek met Mensen)
