# Supplementary material for: Multicenter randomized clinical trial of endovascular treatment for acute ischemic stroke. The effect of periprocedural medication: acetylsalicylic acid, unfractionated heparin, both, or neither (MR CLEAN-MED). Rationale and study design
Source: Trials. 2020 Jul 14;21:644. doi: 10.1186/s13063-020-04514-9 (PMC7362523; doi:10.1186/s13063-020-04514-9)
Supplement: Supplementary file 1 — Additional file 1. MR CLEAN-MED monitoring plan (Dutch). [file 13063_2020_4514_MOESM1_ESM.pdf]

BIJLAGE B
Frequentie en Inhoud Monitoring voor
ONDERZOEK MET MATIG RISICO

FREQUENTIE MONITORING
2-3 visite per jaar per centrum afhankelijk van inclusiesnelheid en eerder geobserveerde deviaties.

INHOUD MONITORING
Studiedocumenten en afspraken
- Controle aanwezigheid en volledigheid van het onderzoeksdossier: Trial Master File en Investigator File.
- Controle instructies studiepersoneel en afspraken over back-up door bevoegde collega's.

Patiënteninstroom, consent, compliance en Source Document Verification (SDV)
- Controle inclusiesnelheid en uitval percentage.
- Controle informed consent: steekproef: 25%.
- Controle in- en exclusiecriteria: steekproef: eerste 3 deelnemers, daarna 25%.
- Controle protocolcompliance: steekproef: eerste 3 deelnemers, daarna 25%.
- Source Document Verification (SDV) steekproef: 25%; wordt uitgevoerd op basis van een van tevoren gedefinieerde lijst van variabelen - inclusief primair eindpunt - die in duidelijke relatie staan tot de veiligheid en geldigheid van het onderzoek.

Patiëntveiligheid
- Controle Serious Adverse Event (SAE) reporting: steekproef 25 % van de proefpersonen.
- Controle Suspect Unexpected Serious Adverse Reaction (SUSAR) reporting: steekproef 25 % van de proefpersonen (indien van toepassing).

Studiemedicatie of onderzoeksproduct (indien van toepassing)
- Controle welke instructies deelnemers meekrijgen.
- Controle overzichten van ontvangst, opslag, uitgifte, retour en voorraadbeheer.
- Controleer aanwezigheid correcte procedure voor deblindering in geval van nood.

Studieprocedures
- Controle of instructies voor uitvoer van studieprocedures aanwezig zijn.
- Controle apparatuur en /of faciliteiten.

Laboratorium, apotheek en biologische monsters (indien van toepassing)
- Controle of laboratoria GLP gecertificeerd zijn.
- Controle of apotheken GMP gecertificeerd zijn.
- Controle verzameling, labeling en opslag van biologische monsters.

AANDACHTSPUNTEN
- **kwalificaties monitor**
- **terugkoppeling en follow-up van bevindingen van de monitor**
  - Termijn van beschikbaarheid monitoring-rapporten.
  - Acties naar aanleiding van verbeterpunten in monitoring rapport: binnen Erasmus MC en in andere deelnemende centra (in geval van multcenter trial).
- **bewaren van studiegegevens**
  - Gebruik van een adequaat Clinical Data Management Systeem (CDMS).
  - Correct bewaren van ruwe gegevens, gecorrigeerde gegevens en back-ups.
  - Beschikbaarheid van een audit trail (herleidbaarheid van studiedata en aanpassingen).

MONITORINGRAPPORTEN EN BEWAARTERMIJN
Van elke monitoringvisit wordt een schriftelijk verslag gemaakt, het monitoringrapport. Het afdelingshoofd van de hoofdonderzoeker is verantwoordelijk voor de archivering van de monitoringrapporten gedurende minimaal 15 jaar na afronding van het onderzoek. De monitoringrapporten en overige onderzoeksdocumenten zijn op verzoek toegankelijk voor de Raad van Bestuur van het Erasmus MC, en voor door de Raad van Bestuur geautoriseerde medewerkers.
